# Intracranial Lipoma Extending Extracranially in a Five-Year-Old Patient

**DOI:** 10.7759/cureus.21816

**Published:** 2022-02-01

**Authors:** Mohamed Abdelgadir M Elgassim, Amin Wafer, Amina Ahmed, Anas Elfaki, Ahmed Satti, Shahzad Anjum

**Affiliations:** 1 Emergency Medicine, Hamad Medical Corporation, Doha, QAT; 2 Emergency Medicine, Hamad General Hospital, Doha, QAT; 3 Pediatrics, Hamad Medical Corporation, Doha, QAT; 4 Dentistry, Hamad Medical Corporation, Doha, QAT

**Keywords:** intra-axial brain tumor, brain tumor, pediatric brain tumor, rare benign tumor, benign brain tumor, intracranial tumor, extracranial lipoma, lipoma, intracranial lipoma

## Abstract

Intracranial lipomas are one of the rarest brain lesions. It is thought to form due to abnormal persistence and differentiation of the meninx. Here we report a unique case of a five-year-old male child with no known chronic medical illnesses and with no history of previous surgeries or allergic problems. He was brought to the pediatric emergency department after having episodes of focal seizures, which lasted only two minutes. On arrival to the emergency department, the child had no neurological deficits or any form of distress. A detailed neurological examination was conducted, and it was normal. Brain CT was requested according to the departmental policy, which showed a well-defined oval shape homogenous fat density in the midline along the falx cerebri at the vertex level, likely representing interhemispheric lipoma. Intracranial lipomas are rare and usually asymptomatic lesions that are formed of adipose tissue. The tumor is usually diagnosed as an incidental finding on CT or MRI scans as patients are usually asymptomatic. However, if symptomatic, the most common presentation of this tumor is seizures. The management is usually conservative, and surgical intervention is not usually recommended.

## Introduction

Intracranial lipomas are rare growths that represent less than 0.1% of all brain tumors [1]. Most of these lipomas are asymptomatic and incidentally found on imaging while assessing other conditions. The most common symptoms intracranial lipomas present with are seizures, headaches, and vertigo [2]. Magnetic resonance imaging (MRI) and computed tomography (CT) scans are both acceptable diagnostic modalities [3]. Here we are presenting a rare case of intracranial lipoma along the midline in the falx cerebri in a five-year-old child who presented with seizure and also discuss the approach and recommended management options.

## Case presentation

**Figure 1:**
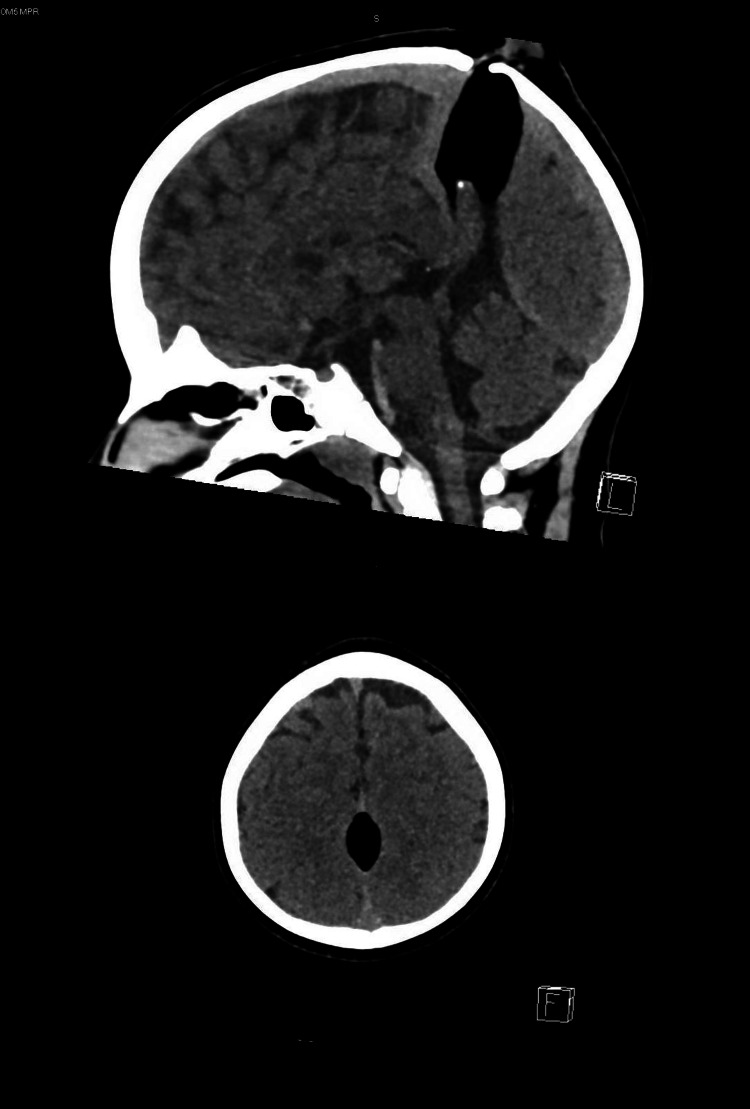
Intracranial lipoma showing extracranial extension

A five-year-old male patient with no underlying medical or surgical problem was brought to the pediatric emergency department because of a single focal seizure which happened 2 hours prior to arrival in the emergency department. The seizure started while he was lying in his father's arms. He suddenly started snoring, his eyes rolled upward, his right upper and lower limbs started jerking heavily, and he was having frothy secretion from his mouth. The episode lasted for 2 minutes and was followed by a period of unresponsiveness for 1 minute after which the child woke up and was responsive. On arrival to the pediatric emergency department, He was not distressed. His vital signs were stable, and he was afebrile. On examination, there were no neurological deficits, but examination of the head revealed a soft central mass with normal skin and no hair on it. The neurologist on-call was consulted, who recommended performing a plain CT of the head as part of the workup for the focal seizure. The CT of the head revealed well-defined oval-shaped homogenous fat density (-111 Hounsfield units [HU]) seen in the midline along the falx cerebri at the level of the vertex. It measured 2.5 x 1.5 x 4 cm in maximum AP x TR x CC [anteroposterior x transverse x craniocaudal] dimensions, likely representing interhemispheric lipoma. No significant mass effect is seen, as shown in Figure 1. He was kept for observation for 8 hours and then discharge on buccal midazolam 7mg as needed for seizures and regular oxcarbazepine 120mg twice a day (BID) for two weeks, 180mg BID for two weeks, 240mg BID for two weeks, and then 300mg BID for six months. On his follow-up referral, his EEG showed frequent left temporal, central, and occasional parietal spikes, with slow spike wave with no generalization. The child’s condition remained stable, and he remained seizure-free two months after the initial episode.

## Discussion

Intracranial lipomas are rare malformations that form due to the abnormal persistence of meninx primitiva, which is the mesenchymal tissue that the meninges arise from and differentiate into adipose tissue. These lipomas are very rare, most commonly idiopathic in origin, and located in the interhemispheric fissure [4,5].

The presenting symptoms for patients with these lipomas are dependent on the location of the tumors intracranially and how they affect the brain. These symptoms may include headaches, seizures, or vertigo, but most commonly the patients are asymptomatic, and these tumors are found as an incidental finding [2]. Our patient has had an occipital swelling since birth and presented to the emergency department due to new onset of seizures for workup.

Intracranial lipomas in the interhemispheric region associated with subcutaneous components are extremely rare, with a handful of cases being reported in the literature [3,6-9]. The two identified types of intracranial lipomas are the tubulonodular and curvilinear types. Tubulonodular types tend to be cylindrical, round, and anterior, with an axial diameter of >1cm. On the other hand, the curvilinear lipomas are usually posteriorly situated, and their length is more than their width, maintaining an axial diameter of <1cm [10]. Our patient had the tubulonodular type of intracranial lipoma, which extends extracranially, in the posterior direction, and forms an occipital scalp swelling, making it a unique case.

Both MRIs and CTs serve as diagnostic tools for intracranial lipomas [3]. MRI can be used for assessing the anatomy, monitoring the tumor growth, and assessing malignancies. However, plain CT scans remain a diagnostic modality only, and the lipomas appear as a homogenous mass with fat density attenuation ranging from -80 to -110 HU [6]. In our case, the CT scan showed a well-defined oval-shaped homogenous fat density (-111 HU).

One of the main limitations to this study is the deficiency in imaging as neither an MRI nor a contrast CT was performed. Nevertheless, the plain CT scan performed as part of a complex seizure workup in the emergency department was sufficiently diagnostic.

The majority of symptomatic intracranial lipomas are managed medically due to the complexity of surgical intervention considering the passage of nerves and blood vessels through the tumor [3]. Furthermore, undergoing surgical resection of the tumor has not been proven to relieve the most common symptom associated with the intracranial lipoma, i.e., seizures, making the risks outweigh the benefits [6]. Further research is needed to study the effect of external trauma or pressure on the extracranial portion of the lipoma causing intracranial pressure fluctuations and leading to CNS symptoms.

## Conclusions

Intracranial lipomas are rare and usually asymptomatic lesions formed of adipose tissue. The tumor is usually diagnosed through incidental findings in CT or MRI scans as patients are usually asymptomatic. However, if symptomatic, the most common presentation is seizures. Management is usually medical through symptomatic treatment, and surgical intervention is not recommended.
